# Medical Image Segmentation Methods: A Decision-Guided Survey Covering 2D/3D CNNs, Transformers, VLMs, SAM-Based Models and Diffusion Approaches

**DOI:** 10.3390/bioengineering13050555

**Published:** 2026-05-15

**Authors:** Kadir Sabanci, Busra Aslan, Muhammet Fatih Aslan

**Affiliations:** 1 Department of Electrical-Electronic Engineering, Karamanoglu Mehmetbey University, Karaman 70100, Türkiye; 2 Department of Electronics and Automation, Vocational School of Technical Sciences, Konya Technical University, Konya 42000, Türkiye; baslan@ktun.edu.tr; 3 Department of Artificial Intelligence and Machine Learning, Faculty of Computer and Information Sciences, Konya Technical University, Konya 42000, Türkiye; mfaslan@ktun.edu.tr

**Keywords:** medical image segmentation, vision-language models, Segment Anything Model (SAM), diffusion models, model selection, domain shift

## Abstract

Recent advances in medical image segmentation have introduced a wide spectrum of deep learning paradigms, including 2D/3D convolutional neural networks (CNNs), transformer-based architectures, vision-language models (VLMs), prompt-driven foundation models such as Segment Anything Model (SAM), and diffusion-based approaches. Although these methods have demonstrated remarkable performance across MRI, CT, PET, ultrasound, and endoscopic imaging, the rapid proliferation of architectures has created methodological uncertainty regarding optimal model selection under varying clinical and data constraints. Existing surveys primarily focus on architectural categorization, yet provide limited guidance for decision-oriented model selection. This study presents a comprehensive and decision-guided survey that systematically analyzes segmentation paradigms across imaging modalities, task types, dataset characteristics, and evaluation protocols. Beyond taxonomy, we propose a practical model selection framework that links clinical scenarios, such as small lesion detection, multi-organ 3D segmentation, limited-data regimes, and domain shift, to appropriate segmentation strategies. Furthermore, robustness, generalization, annotation variability, and benchmarking reproducibility are critically examined. By integrating architectural taxonomy, cross-modal comparative analysis, and a structured decision framework, this work provides a clinically oriented roadmap for selecting segmentation methods and highlights future research directions toward reliable and reproducible medical AI systems.

## 1. Introduction

Medical image segmentation constitutes the foundation of imaging-based clinical decision support systems by enabling the accurate delineation of organs, tissues, or pathological structures. In particular, the emergence of deep learning approaches has initiated a new era in the field by replacing traditional methods [1]. Convolutional Neural Network (CNN)-based models, which pioneered this process, have achieved high accuracy by effectively capturing local spatial features; in particular, the U-Net architecture has been a milestone in the field of segmentation due to its encoder-decoder structure [2]. Classical 2D CNNs face limitations when processing volumetric data, which have been partially addressed by architectures such as 3D U-Net [3]. However, the inherent nature of convolution operations fails to adequately represent global contextual relationships (long-range dependencies). Consequently, this shortcoming continues to limit model performance in complex anatomical structures [4,5]. These limitations have led to the adaptation of Transformer-based architectures to medical image analysis; models such as TransUNet [5] and Swin-UNet [4] have succeeded in going beyond CNNs with their ability to learn long-range dependencies [6].

In recent years, the adaptation of large-scale foundation models and particularly prompt-based approaches such as the Segment Anything Model (SAM) to the medical domain has brought methodological diversity to an unprecedented level [7,8]. However, this rapid evolution from task-specific traditional models to multi-purpose interactive models leads to significant uncertainty for researchers and clinical practitioners regarding which model to select for a specific data type or clinical scenario. This situation, defined in the literature as “decision paralysis,” becomes even more complex when considering different imaging modalities (e.g., MRI, CT, ultrasonography), dataset size limitations, annotation quality, and hardware constraints [1,9]. Therefore, in today’s research ecosystem, not only the development of new models but also the establishment of a systematic decision support framework to guide optimal model selection within this vast body of literature has become a critical requirement from both clinical and academic perspectives.

### 1.1. Why Segmentation Remains the Core Task in Medical Imaging

Although various computer vision tasks such as classification, object detection, and image generation exist in medical imaging analysis, segmentation remains the most fundamental and indispensable step in the clinical workflow [10]. The primary reason for this is that segmentation is the only method capable of quantitatively revealing the morphological properties (volume, shape, boundaries, and spatial relationships) of anatomical structures and lesions.

While classification algorithms provide only image-level information such as “presence or absence of a tumor” or “benign/malignant,” segmentation algorithms present actionable information to the clinician regarding “the exact location of the tumor, the extent of its spread, and its boundaries with surrounding healthy tissues.” For example, in oncological imaging, precise measurement of tumor size and volume is a clinical requirement for disease staging and monitoring treatment response according to RECIST (Response Evaluation Criteria in Solid Tumors) protocols [11].

Similarly, segmentation plays a critical role in radiotherapy planning. Delivering the radiation dose at a maximum level to the targeted Gross Tumor Volume while protecting surrounding healthy tissues (Organs-at-Risk) from radiation is only possible through precise voxel-level segmentation on 3D CT or MRI slices. In addition, patient-specific surgical navigation and robotic intervention systems also require precomputed high-resolution 3D maps of organs and lesions [12].

For these reasons, although deep learning paradigms have evolved from traditional CNNs to large-scale Vision-Language and foundation (e.g., SAM) models, segmentation, which is the art of clinically interpreting pixels, continues to remain at the core of diagnosis and treatment processes.

### 1.2. Objectives and Contributions of This Decision-Guided Survey

Several recent surveys have examined deep learning-based medical image segmentation from complementary perspectives. Gao et al. [1] provide a broad review of deep learning methods across modalities, offering valuable guidance on model selection by detailing applicable scenarios, limitations, data dependencies, and hardware constraints. Yao et al. [6] trace the architectural evolution from CNNs to Transformers with commendable depth, and notably incorporate the foundation model paradigm by evaluating the SAM alongside them. Ali et al. [7] and Takahashi et al. [9] focus specifically on SAM-based models and the CNN-versus-Transformer comparison, respectively, providing valuable but inherently focused perspectives. Zhou et al. [13] offer a broad, forward-looking perspective on the foundation model era in general image segmentation, yet their scope extends beyond medical imaging and does not exclusively translate these architectural insights into practical decision criteria tailored for specific clinical scenarios. While these existing works have made significant contributions in linking model characteristics to practical deployment parameters, there remains an opportunity to integrate these insights into a fully unified, cross-paradigm evaluation framework. The present survey builds upon this foundation by proposing a structured, evidence-based decision framework that systematically compares CNNs, Transformers, and various foundation models to answer which segmentation paradigm should be preferred under which conditions. The reliability of this framework is grounded in a systematic cross-analysis of benchmark results across BraTS, LiTS, KiTS, and MSD datasets, augmented by a critical examination of evaluation metric sensitivity, annotation variability, and reproducibility issues, factors that we argue are central determinants of clinical validity rather than secondary methodological concerns.

The field of medical image segmentation has undergone a major transformation over the past decade under the influence of deep learning-based models. However, the rapid proliferation of methods has led to a lack of systematic guidance for researchers regarding which model to prefer in a specific clinical or technical scenario. This study goes beyond existing surveys and aims to present a decision-oriented evaluation framework in the context of clinical applicability, not only classifying methods but also guiding model selection.

The scope of the study includes a broad methodological spectrum ranging from classical approaches based on CNN to advanced architectures based on Transformers and Vision-Language Models (VLM), as well as the SAM and diffusion-based generative models. The examined models are evaluated in terms of their performance in 2D and 3D segmentation tasks, multimodal data scenarios, small lesion detection, and their generalization capabilities under domain-shift conditions.

In this context, the main contributions of our study to the literature are as follows:

• Holistic Taxonomy: Existing model families (CNN, Transformer, VLM, SAM, and Diffusion) are consolidated under a comprehensive taxonomy and analyzed comparatively in terms of their structural differences, learning strategies, and clinical application areas.
• Modality- and Task-Oriented Comparative Analysis: The strengths and weaknesses of segmentation algorithms are examined across different imaging modalities such as MRI, CT, PET, ultrasonography, and endoscopy; clinical reliability, robustness, and generalization capability are systematically evaluated.
• Decision Support Framework: By considering practical parameters such as data size, modality type, lesion size, presence of domain shift, and computational constraints, an actionable guideline is presented regarding which model should be preferred in which clinical scenario.
• Performance and Transparency Analysis: The sensitivities of different evaluation metrics (Dice, IoU, HD95), the quality of public datasets, and reproducibility issues in the literature are addressed, and standards for the reliability of clinical AI systems are proposed.

### 1.3. Literature Search Strategy

**Figure 1:**
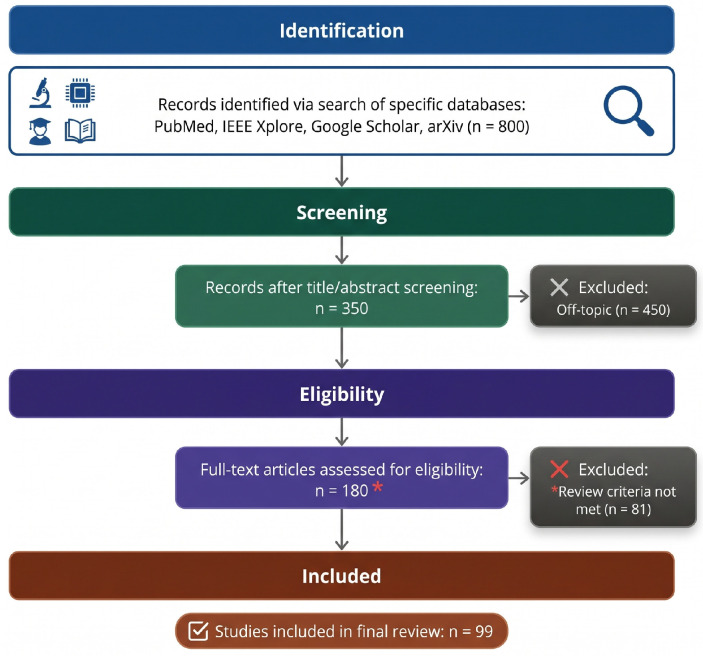
PRISMA-style flow diagram of the literature search and study selection process. * Articles excluded at the eligibility stage did not meet the predefined review criteria.

The present survey was conducted through a systematic search of four major academic databases: PubMed, IEEE Xplore, Google Scholar, and arXiv. Searches were performed using the following keyword combinations: “medical image segmentation deep learning”, “CNN segmentation MRI CT”, “transformer medical segmentation”, “segment anything model medical”, “vision language model segmentation”, “diffusion model medical imaging”, and “foundation model segmentation”. The search was restricted to publications from 2015 to 2025, corresponding to the period spanning the introduction of the U-Net architecture to the present day. Inclusion criteria were: (1) peer-reviewed journal articles or high-citation arXiv preprints; (2) direct and substantive contribution to medical image segmentation; (3) publications in English. Exclusion criteria were: (1) studies with only indirect relevance to segmentation; (2) works lacking sufficient experimental evaluation; (3) studies focused exclusively on hardware or clinical protocol design without algorithmic contribution. From an initial pool of 800 records, 350 were retained after title and abstract screening, and 180 underwent full-text assessment. Following the application of inclusion and exclusion criteria, 99 studies were included in the final review. The paper selection process is summarized in Figure 1 in the style of a PRISMA flow diagram.

### 1.4. Structure of the Paper

This review article is structured to examine existing methods in the field of medical image segmentation through a systematic and decision-oriented approach and is designed in a multi-layered manner to encompass both theoretical foundations and application-oriented implications. The remainder of the study is organized as follows. Section 2 presents the fundamental conceptual framework related to medical imaging modalities, segmentation tasks, data characteristics, and common evaluation metrics. Section 3 provides a detailed architectural taxonomy ranging from classical CNNs to Transformers, Vision-Language models, SAM derivatives, and diffusion-based approaches. Subsequently, Section 4 addresses open-access datasets and benchmarking protocols in the context of annotation quality. Section 5 discusses comparative performance analyses across different modalities and tasks in terms of accuracy, generalization, and computational cost. Section 6 represents the core contribution of this article by introducing an actionable decision support framework that answers the question, “which model should be preferred under which conditions?”. Furthermore, Section 7 examines the dimensions of clinical reliability, uncertainty estimation, and robustness against domain shift. Section 8 highlights future research directions, such as the adaptation of foundation models and federated learning. Finally, Section 9 concludes the review by summarizing the overall findings.

**Figure 2:**
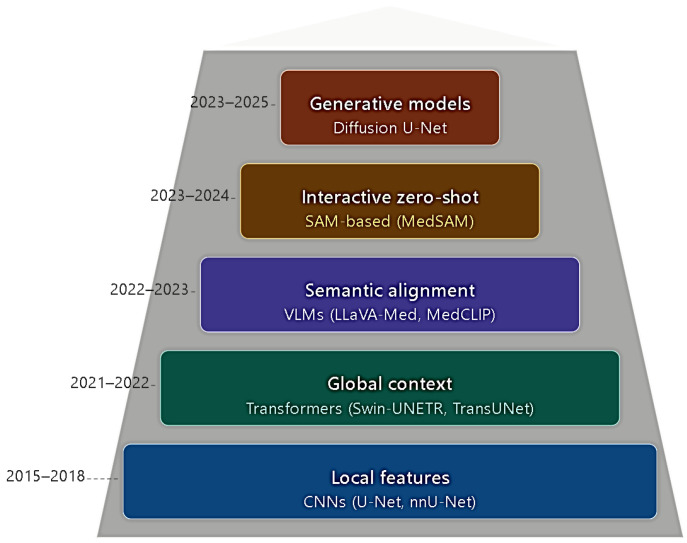
Architectural evolution in medical image segmentation. The figure illustrates the chronological progression of segmentation paradigms from local feature extraction via CNNs (2015–2018), through global context modeling via Transformers (2021–2022), semantic alignment via VLMs (2022–2023), interactive zero-shot segmentation via SAM-based models (2023–2024), to generative synthesis via diffusion-based approaches (2023–2025).

**Figure 3:**
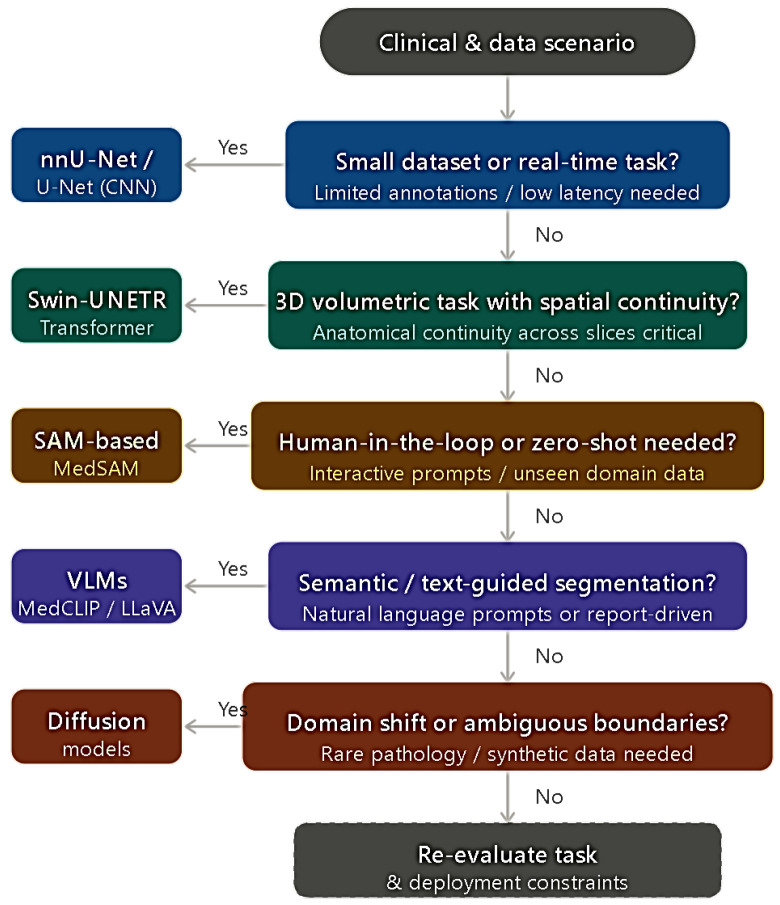
Decision framework: when to use which model? The figure presents a condition-driven decision support tool that guides practitioners through a sequential series of clinical and data scenario nodes, directing toward the most appropriate segmentation paradigm, namely CNNs, Transformers, SAM-based models, VLMs, or diffusion models, based on dataset size, volumetric continuity, generalization, and deployment constraints.

The architectural evolution described throughout this review, from local feature extraction via CNNs to generative synthesis via diffusion-based approaches, is visually summarized in Figure 2. Each tier in the figure corresponds to a distinct methodological shift discussed in Section 3, allowing readers to situate individual architectures within the broader historical and conceptual progression of the field. The corresponding decision support framework, which guides model selection across clinical and data scenarios as proposed in Section 6, is presented in Figure 3. The framework should be read as a sequential decision tree, where each node represents a binary clinical or data condition that directs the practitioner toward the most appropriate segmentation paradigm for their specific deployment context.

## 2. Background and Problem Definition

### 2.1. Medical Image Modalities and Segmentation Tasks (MRI, CT, PET, US, Endoscopy, Dermoscopy)

Medical imaging is one of the most important components of clinical decision support systems, and image segmentation constitutes the most critical sub-stage of this process. Segmentation aims to automatically delineate biological structures such as organs, tissues, or lesions from images. Unlike natural images, medical images contain unique challenges such as low contrast, complex overlapping anatomical boundaries, and high noise levels [14]. Today, the main modalities most commonly used in medical image segmentation are Magnetic Resonance Imaging (MRI), Computed Tomography (CT), Positron Emission Tomography (PET), Ultrasonography (US), and endoscopic/dermoscopic images [1]. Each of these modalities requires specific segmentation strategies due to their different contrast characteristics and noise profiles:

• MRI (Magnetic Resonance Imaging): It is a method that provides high-contrast imaging of soft tissues and does not involve ionizing radiation. It is widely used in the segmentation of the brain, musculoskeletal system, cardiac, and abdominal organs. The main strength of MRI lies in its ability to provide complementary information about tissues (e.g., anatomical structure, edema, and necrotic core) by offering multi-parametric sequences such as T1, contrast-enhanced T1 (T1c), T2, and FLAIR [15]. However, the high variance intensity distributions of MRI data and motion artifacts arising from long acquisition times are factors that complicate model generalization. The BraTS (Brain Tumor Segmentation Challenge) dataset has become a global standard for deep learning models in MRI segmentation [16].
• CT (Computed Tomography): It is an imaging technique based on ionizing radiation and is preferred for high-resolution 3D analysis of bones, lungs, blood vessels, and abdominal organs. Large-scale benchmarks such as LiTS (Liver) and KiTS (Kidney) are widely used in CT segmentation research [17,18]. Although the high spatial resolution of CT data provides a significant advantage in delineating anatomical boundaries, accurately defining the boundaries of soft tissue lesions with very similar Hounsfield unit (HU) values remains an important challenge for deep learning models.
• PET (Positron Emission Tomography): It contains functional information representing metabolic activity rather than anatomy. It is used particularly in oncological imaging for tasks such as the detection of tumors with high FDG uptake and the monitoring of metastasis [19]. Due to the nature of PET data, spatial resolution is very low and lesion boundaries are blurred. Therefore, PET images are often combined with CT or MRI (PET/CT or PET/MRI) and fed into multimodal segmentation networks. In this way, the functional (metabolic) sensitivity of PET is combined with the anatomical precision of CT to obtain precise lesion maps [20,21].
• US (Ultrasonography): It is one of the most widely used methods in clinical practice due to its portability, low cost, and real-time imaging capability. It is frequently preferred in fetal, cardiac, breast, and liver examinations. However, speckle noise inherent in US data, low tissue contrast, shadowing artifacts, and high operator dependency (probe holding angle) are major obstacles to the production of stable results by segmentation algorithms [22].
• Endoscopic and Dermoscopic Images: They provide high-resolution optical (RGB) visualization of internal organ lumens and the skin surface. The ISIC dataset, used for the detection of skin lesions (e.g., melanoma), and the CVC-ClinicDB or Kvasir-SEG datasets, which enable polyp detection in colonoscopy, are the cornerstones of this field [23,24]. The main challenges encountered by segmentation in these images include occlusion by surgical instruments, variable illumination conditions, reflections, and the low color contrast between lesions and the background tissue (camouflage effect).

The diversity of these modalities necessitates that segmentation models develop architecture-specific adaptations for each data type. Today, instead of relying on a single modality to overcome these challenges, multimodal fusion techniques in which images complement each other (e.g., CT + PET) and robust transfer learning approaches that compensate for distribution shifts (domain shift) in data from different devices/centers are emerging [9]. Thus, by combining the modality-specific clinical strengths of each modality, more robust decision support systems are constructed.

### 2.2. 2D vs. 3D Segmentation: Fundamentals and Challenges

Deep learning approaches used in medical image segmentation are classified as two-dimensional (2D), three-dimensional (3D), and hybrid (2.5D) architectures according to the spatial dimensionality of the data and the processing strategy. This distinction is not only related to the format of the input data; it is also a fundamental design choice that directly determines the model’s ability to capture spatial context, hardware cost, and clinical accuracy.

Dynamics and Limitations of 2D Architectures: 2D approaches (e.g., standard U-Net) [2] process 3D volumetric data such as MRI or CT as independent 2D slices (typically axial sections). The main advantage of these models is their low computational cost, high inference speed, and the ability to easily utilize pre-trained weights from the natural image domain through transfer learning. However, these methods suffer from discontinuity issues in 3D space because they ignore the anatomical relationships between consecutive slices (inter-slice information) [25]. In particular, the loss of volumetric consistency in 3D structures with irregular and complex boundaries, such as brain tumors or liver lesions, is the main drawback of 2D models.

Power and Bottlenecks of 3D Architectures: 3D approaches developed to address this limitation (e.g., 3D U-Net [3], V-Net [26]) process the entire volumetric data or multiple patches (3D patches) simultaneously using 3D convolutions. In this way, inter-slice context and organ integrity are preserved, and higher anatomical accuracy is achieved. However, 3D models have significant disadvantages: due to the increased processing dimensionality, they require large GPU memory and have longer inference times [27,28]. More importantly, the enormous number of parameters in 3D architectures exposes the model to a significant risk of overfitting in medical datasets where labeled data is limited.

Anisotropic Data Problem and 2.5D Solutions: The anisotropic voxel problem frequently encountered in clinical scans is the most important factor that complicates the choice between 2D and 3D approaches. In most medical images, while the resolution in the x and y axes is high, the resolution in the z axis (slice thickness) is low. In such highly anisotropic data, directly using 3D CNNs may reduce performance by causing the network to learn irrelevant and misleading features along the z-axis [28,29]. To overcome these challenges, 2.5D segmentation (pseudo-3D) approaches have been developed [30]. 2.5D models integrate 3D spatial information with much lower computational cost by combining predictions from 2D slices obtained from different orthogonal planes (axial, coronal, sagittal) (multi-view fusion) or by providing consecutive slices to the model as multi-channel inputs (multi-slice input).

As a result, the choice of architecture depends on the isotropic/anisotropic nature of the data, the volumetric complexity of the target organ, and the available hardware constraints. Today, automatic configuration systems such as nnU-Net analyze dataset characteristics (image size, voxel spacing, class balance) and, based on hardware constraints, automatically select the most suitable option among 2D, 3D full-resolution, or 3D cascade (low-res to high-res cascade) architectures using empirical rules, thereby optimizing this trade-off [31].

### 2.3. Evaluation Metrics Overview

Evaluating the performance of methods developed in the field of medical image segmentation is critical not only for achieving high accuracy, but also for ensuring that results are reproducible and clinically meaningful. In this context, different classes of metrics have been developed to objectively assess segmentation outcomes: overlap-based, distance-based, and lesion-wise metrics [32,33].

The most widely used overlap-based metric is the Dice Similarity Coefficient (DSC). First defined by Dice (1945) [34], this coefficient measures the similarity between the predicted region and the ground truth region:

$$DSC=\frac{2\left|A\cap B\right|}{\left|A\right|+\left|B\right|}$$

where *A* and *B* represent the predicted and reference masks, respectively. As the Dice coefficient approaches 1, the degree of overlap increases. However, class imbalance and the presence of small lesions may reduce the sensitivity of classical pixel-based overlap metrics [33].

The Intersection over Union (IoU) metric, also known as the Jaccard Index, represents the ratio of the intersection of two sets to their union:

$$IoU=\frac{\left|A\cap B\right|}{\left|A\right|\cup \left|B\right|}$$

It has been demonstrated in the literature that IoU and Dice are mathematically interconvertible, with

$$IoU=\frac{Dice}{2-Dice}$$

and yield identical results when ranking methods [32,33]. Although IoU is a standard metric in general computer vision tasks, DSC has traditionally been preferred in medical imaging.

Among the metrics that measure geometric accuracy, the Hausdorff Distance (HD) computes the maximum distance between the boundaries of the predicted and ground truth segmentations. However, its 95th percentile variant (HD95) is commonly preferred to avoid the influence of a single outlier value. This metric provides a more robust performance measurement in the presence of noise and local errors [35].

Another important metric, the Average Symmetric Surface Distance (ASSD), evaluates boundary smoothness by measuring the mean mutual distance between two surfaces. This measure reveals geometric and boundary deviations that volume-based metrics such as Dice may overlook [33].

### 2.4. Common Preprocessing Pipelines

In the field of medical image segmentation, the success of deep learning models depends not only on the capacity of network architectures, but also on the level of standardization of the input data. Since raw data are typically acquired in DICOM format, discrepancies arise in terms of image resolution, orientation, and intensity values. For this reason, modern pipelines first apply DICOM-to-NIfTI conversion, followed by intensity normalization, bias field correction, and registration (image alignment) steps [36].

DICOM-to-NIfTI conversion reduces the heterogeneity arising from multi-vendor data sources. Although the DICOM format contains metadata including device parameters, deep learning models cannot directly process this information. The NIfTI standard represents the data volume as a single 3D tensor, thereby facilitating automated loading and resampling operations in large datasets such as brain (BraTS) or liver (LiTS) benchmarks. This conversion constitutes a fundamental preprocessing step in open-source deep learning frameworks such as NiftyNet [36].

Intensity normalization is applied to reduce signal variations arising from different MR or CT scanners and patient populations. The most common approaches include min-max scaling based on physical units (Hounsfield Units) with clipping for CT images, z-score standardization for MR images [31], as well as MR-specific advanced techniques such as histogram matching and White Stripe [37]. This process stabilizes the model’s learning process and minimizes the impact of contrast differences.

Bias field artifacts observed in magnetic resonance images (that is, low-frequency intensity fluctuations) can significantly impair segmentation accuracy. The N4ITK algorithm [38], developed to correct this problem, homogenizes the signal distribution by modeling the bias field. This step substantially improves performance in intensity-based networks such as U-Net and V-Net, particularly in tasks such as brain MR segmentation.

Image registration, which refers to the spatial alignment of images, enables the same anatomical region to become comparable across different modalities (e.g., MR, CT, PET). Advanced Normalization Tools (ANTs) is one of the most reliable software packages developed for this purpose [39]. ANTs minimize inter-structural differences using deformable registration methods and enhances the anatomical accuracy of segmentation masks. This approach enables the spatial standardization of data obtained from multi-center studies.

Scanner and field strength differences in data collected from multiple centers also constitute a significant standardization challenge. Harmonization approaches developed by Fortin et al. [40] (e.g., ComBat) statistically mitigate scanner-induced variations, thereby improving the overall performance of the model. Such harmonization procedures are of critical importance for enhancing the generalizability of segmentation models, particularly in multi-site clinical studies.

Ultimately, a successful segmentation system is defined not only by its network architecture, but also by the quality of data preprocessing. The standardization of processes such as DICOM to NIfTI conversion, bias correction, normalization, and registration ensures that model performance is both reproducible and clinically reliable.

### 2.5. Challenges Intrinsic to Medical Data (Annotation Scarcity, Class Imbalance, Domain Shift)

Due to the inherent nature of medical image data, there are fundamental challenges that limit the performance of deep learning models. In particular, the problems of annotation scarcity, class imbalance, and domain shift directly affect both the accuracy and clinical generalizability of models [14,41].

Annotation scarcity is the most prevalent structural problem in medical image segmentation. Since the labeling of images can only be performed by expert radiologists or pathologists, this process is highly time-consuming and costly. Furthermore, observer-dependent interpretive variability during the annotation process introduces noise into the model. Consequently, access to large-scale, high-quality labeled datasets is often not feasible [14]. To address this deficiency, semi-supervised, self-supervised, and transfer learning approaches are widely employed in the literature [41]. These methods enable models trained on limited labeled data to learn more generalizable representations by transferring knowledge from unlabeled data or data acquired across different modalities.

Class imbalance arises from the fact that lesions or small anatomical structures that are structurally rare in medical images occupy a substantially smaller pixel ratio compared to large background tissues. This imbalance introduces a background bias in model predictions and leads to the neglect of small pathological regions. To address this problem, class-weighted loss functions such as focal loss and Dice loss have been developed. In addition, GAN (Generative Adversarial Network)-based data augmentation methods have been employed to enhance the representational power of minority classes. Han et al. [42] performed synthetic data generation for brain MR tumor detection by combining noise-to-image and image-to-image GANs. Similarly, Frid-Adar et al. [43] applied GAN-based data augmentation in liver lesion classification to mitigate the impact of imbalanced class distributions on model performance. These studies demonstrate that data-driven solutions can be effective against class imbalance.

The third challenge, domain shift, arises from differences in intensity distributions, contrast, resolution, and noise profiles among images acquired from different clinical centers, device manufacturers, or patient populations [44]. When a model is trained on data with a specific distribution, it experiences performance degradation on samples from a different data source. This situation leads to serious generalizability problems, particularly in multi-center clinical studies. Domain adaptation approaches have been developed as a solution to this problem. The test-time adaptation method proposed by Karani et al. [45] allows the model to adapt itself to new data distributions during inference. Such adaptive models dynamically minimize variations arising from domain discrepancies.

To summarize, the structural nature of medical data (limited annotations, imbalanced class distributions, and discrepancies across data sources) constitutes the three primary factors restricting the clinical validity of deep learning-based segmentation models. Consequently, modern research trends focus not only on architectural innovations, but also on learning strategies robust to data scarcity, balancing data augmentation, and adaptive modeling approaches [14,45].

## 3. Taxonomy of Medical Image Segmentation Methods

### 3.1. Classical CNN-Based Architectures

Classical CNN-based segmentation architectures constitute the foundational structures that initiated the rise of deep learning in the field of medical image analysis. These architectures have evolved primarily through the U-Net family and have established the de facto standards of the field. Originally introduced by Ronneberger et al. [2], U-Net revolutionized biomedical image segmentation through its encoder-decoder architecture. By virtue of skip connections that integrate low-level spatial information with high-level semantic representations, this architecture enables high accuracy to be achieved even with limited data. Although U-Net was initially developed for microscopic cell segmentation, it was rapidly adapted to a wide variety of modalities including MR, CT, and fundus imaging [2].

3D U-Net, the volumetric extension of U-Net to three-dimensional data, was proposed by Çiçek et al. [3]. This model replaces 2D convolutions with 3D convolutions, thereby preserving the three-dimensional context of volumetric medical images (e.g., MRI, CT). The preservation of anatomical continuity through 3D context has provided a substantial advantage particularly in tasks such as brain, liver, and prostate segmentation.

Similarly, V-Net, developed by Milletari et al. [26], targeted the class imbalance problem in medical segmentation by introducing a loss function based on the Dice coefficient (Dice Loss). V-Net combined a 3D CNN architecture with residual blocks, thereby facilitating the training of deeper networks and improving accuracy particularly on brain MRI data. This evolution in CNN architectures subsequently reached its apex with approaches such as UNet++ [46], which densifies skip connections, and nnU-Net [31], which automatically configures the architecture according to data characteristics.

Overall, classical CNN-based architectures, particularly U-Net and its variants, constitute the building blocks of modern medical segmentation. These architectures have also served as the foundation for subsequently developed transformer-based or multimodal approaches.

### 3.2. Transformer-Based Architectures

CNN-based approaches have been employed as the dominant architectures in medical image segmentation for many years. However, the limited receptive field of these models has constrained the modeling of long-range dependencies. Transformer-based architectures, developed to address this deficiency, have introduced a new paradigm in medical image segmentation by enhancing global context awareness [5,6].

The transformer concept gained prominence in the field of computer vision with the Vision Transformer (ViT) model [47]. ViT divides an image into fixed-size patch units, processes each as a token, and learns the relationships among them through a self-attention mechanism. This approach, by virtue of its ability to model global relationship networks within an image (in contrast to the local filters employed in CNNs), has established a powerful foundation for medical applications.

The first successful adaptation of the transformer architecture in medical segmentation was achieved with the UNETR (U-Net Transformer) model [48]. UNETR combines low-level spatial information with high-level semantic representations by employing pure transformer blocks in the encoder and CNN-based structures in the decoder. Notably, a higher accuracy compared with classical U-Net was achieved, particularly on the BraTS brain tumor dataset. Extending this architecture, Swin-UNETR [49] integrates both local and global information in a multi-scale manner through a shifted window attention mechanism. This approach has demonstrated superior generalizability compared to UNETR on the Medical Segmentation Decathlon (MSD) datasets.

TransUNet [5], introduced a notable innovation by hybridizing CNN and Transformer architectures. While the CNN module captures local textural information, the ViT-based module learns global contextual relationships. As a result, TransUNet has demonstrated superior performance over CNN-based methods in abdominal organ and cardiac segmentation tasks. Building on a similar hybrid philosophy but targeting volumetric data, nnFormer [50] is designed for volumetric (3D) segmentation and integrates multi-scale features through an interleaved attention mechanism. The model has distinguished itself particularly in volumetric tasks such as brain, liver, and kidney segmentation through attention-based context modeling.

Overall, transformer-based architectures have substantially advanced the state of the art in medical image segmentation by overcoming the inherent limitations of CNN-based models in capturing long-range contextual dependencies. From pure transformer encoders to hybrid CNN-Transformer designs, these models have demonstrated consistent improvements across diverse segmentation benchmarks. Nevertheless, their high computational cost and large parameter counts remain practical challenges, particularly in resource-constrained clinical settings. This has motivated subsequent research toward more efficient attention mechanisms and foundation model-based approaches that retain global context awareness while improving scalability.

### 3.3. Vision-Language Models (VLMs) for Segmentation

One of the most significant innovations observed in medical image segmentation in recent years is the rise of Vision Language Model (VLM)-based approaches. By integrating visual and linguistic information within a joint embedding space, these models enable the semantic interpretation of medical images through natural language descriptions. This allows for segmentation that can be guided at the semantic level, beyond classical pixel-based methods [51]. The foundation of VLMs was established with the Contrastive Language-Image Pretraining (CLIP) architecture. Proposed by Radford et al. [52], CLIP was trained on 400 million image-text pairs through contrastive learning, optimizing the semantic alignment between images and their descriptions. By virtue of its zero-shot learning capability, this architecture has demonstrated high generalizability even on classes not encountered during training.

One of the adaptations of the CLIP paradigm to the medical domain is MedCLIP. Wang et al. [53] trained the model on image–report pairs obtained from the MIMIC-CXR and CheXpert datasets, constructing a visuo-textual representation sensitive to medical concepts. Similarly, the PMC-CLIP [54] model proposed a general-purpose biomedical CLIP model by utilizing millions of article–figure pairs from the PubMed Central (PMC) database. These models have demonstrated the potential to directly associate medical texts with visual segmentation operations in text-guided segmentation tasks. A more advanced version of this architecture is the LLaVA-Med model. Developed by Li et al. [55], this model integrates a CLIP-like visual encoder with a large language model (LLM), thereby acquiring the capability to directly map natural language inputs to relevant anatomical regions in medical images. As a result, it has achieved high performance in prompt-based segmentation tasks guided by user prompts (e.g., “highlight the pulmonary lesion”).

However, several practical challenges remain unresolved. The alignment between natural language descriptions and pixel-level segmentation masks is particularly difficult in medical contexts, where clinical terminology is often ambiguous, highly specialized, or institution-specific. Furthermore, the pretraining of VLMs on large-scale medical image-text pairs requires substantial computational resources and access to curated clinical datasets, which are rarely available outside major academic medical centers. These constraints currently limit the real-world deployability of VLM-based segmentation systems and represent a critical direction for future research, particularly toward the development of lightweight, domain-adaptive VLMs capable of operating effectively under limited data and compute conditions.

Therefore, VLM-based models such as CLIP, MedCLIP, PMC-CLIP, and LLaVA-Med have rendered medical image segmentation more flexible, semantically rich, and clinically meaningful by providing multimodal awareness that extends beyond classical CNN- or ViT-based methods [51]. The integration of natural language guidance into segmentation pipelines marks a fundamental shift in how anatomical and pathological structures are delineated, moving from purely visual pattern recognition toward semantically informed, clinician-interpretable decision making. As these models continue to scale and incorporate domain-specific pretraining, their potential to support interactive, report-driven segmentation workflows in real-world clinical environments becomes increasingly compelling.

### 3.4. SAM-Based Segmentation in Medicine

SAM [8] is a pioneering work that initiated the rise of foundation models in visual segmentation. SAM is capable of segmenting objects in any image based on simple prompt inputs provided by the user, such as points, bounding boxes, or text. In contrast to classical task-specific networks, this architecture offers a generalizable artificial intelligence framework trained on large-scale data. With the adaptation of this model to the field of medical imaging, MedSAM [10] emerged. Published in Nature Communications, this work extended SAM’s architecture to different modalities such as CT, MRI, and fundus imaging, eliminating the need for retraining on new data through its zero-shot segmentation capability. MedSAM has demonstrated remarkable performance particularly in learning with low-labeled data.

Various variants of the SAM architecture were rapidly developed. For instance, EviPrompt [56] reduced the model’s dependence on human intervention and mitigated the domain shift problem by enabling automatic prompt generation (auto-prompting) in medical images without requiring additional training. I-MedSAM [57], on the other hand, adopted an implicit representation learning approach, enabling the model to generate denser and sharper segmentation masks in the visual feature space.

Building on these advances, MedSAM-2 [58] extended the SAM architecture to the domain of video-sequence segmentation, achieving a significant performance improvement in time-series-based analysis of 2D/3D medical images. Additionally, Nazzal et al. [59], proposed a practical method that enhances the accuracy of SAM-based networks through test-time augmentation (TTA). This work improved the robustness and clinical generalizability of the model, making it more reliable in real-world scenarios.

Beyond these architectural advances, several practical challenges warrant explicit attention. SAM-based models exhibit a notable sensitivity to prompt quality and placement, where minor variations in point or bounding box inputs can produce substantially different segmentation outputs, introducing user-dependent variability that is difficult to standardize in routine clinical practice. Additionally, the inherently 2D nature of the original SAM architecture poses significant challenges for 3D volumetric segmentation tasks, where maintaining anatomical continuity across slices is a clinical requirement. Addressing these limitations through automatic prompt generation, volumetric extension, and domain-specific fine-tuning represents a critical frontier for future research in this paradigm.

Consequently, SAM and its variants have become the most prominent representatives of the foundation model paradigm in medical image segmentation. The rapid proliferation of SAM-based adaptations (ranging from zero-shot modality transfer in MedSAM, to auto-prompting in EviPrompt, implicit feature learning in I-MedSAM, and temporal extension in MedSAM-2) reflects a broader shift toward prompt-driven, annotation-efficient segmentation frameworks. With their capacity for label-independent learning, domain generalization, and interactive segmentation, these models hold considerable potential to serve as core components of future multimodal clinical systems, provided that challenges related to fine-grained boundary delineation and domain-specific adaptation are further addressed.

### 3.5. Diffusion-Based Segmentation Models

Diffusion-based models represent the most notable generative foundation model approaches of the recent period in medical image segmentation. These models are capable of modeling complex data distributions through noise addition and denoising processes. In particular, the Diffusion U-Net architecture, based on the denoising score matching principle, offers a powerful framework for both image synthesis and pixel-level segmentation [60]. Peng et al. [61] demonstrated that diffusion-based image translation enhances domain adaptation performance in semantic segmentation tasks. By incorporating segmentation labels into the diffusion process through a label guidance approach, this work reduced structural distortions and reinforced the robustness of U-Net-like diffusion networks.

The Prompt-to-Polyp model, proposed by Chaichuk et al. [62], introduced a text-conditioned diffusion system capable of generating polyp images from medical text prompts using the Stable Diffusion framework. Consequently, this approach supports training in endoscopic segmentation scenarios with scarce labeled data by enabling synthetic data generation from textual descriptions. Extending this line of inquiry to a broader scope, Zhan et al. [63] conducted a comprehensive review emphasizing that conditional diffusion models, built upon U-Net architectures, offer high accuracy in both segmentation and image synthesis tasks. Complementing these findings, Chen et al. [64] demonstrated the applicability of score-based diffusion models to dynamic tasks such as deformation correction and motion correction in MR and parallel MRI scenarios. Building further on the controllability of diffusion-based synthesis, Bhattacharya et al. [65] proposed the RadGazeGen architecture, which integrates additional control signals, including radiomics features and radiologist eye-gaze guidance, into the diffusion process to generate high-fidelity, structurally steerable synthetic medical images. Collectively, these advanced synthesis approaches hold considerable potential to mitigate fairness and domain generalization challenges, thereby improving the accuracy of downstream segmentation and lesion detection tasks.

Despite these promising directions, diffusion-based segmentation models face substantial practical challenges that currently limit their clinical applicability. The multi-step denoising process inherent to these architectures results in considerably longer inference times compared to CNN or Transformer-based models, rendering them impractical for time-sensitive clinical scenarios such as intraoperative guidance or real-time endoscopic segmentation. Furthermore, the boundary between generative synthesis and discriminative segmentation remains insufficiently defined in the current literature, raising questions about the clinical interpretability and reliability of diffusion-generated segmentation outputs. Addressing these challenges, particularly through the development of accelerated sampling strategies and hybrid discriminative–generative architectures, constitutes an important direction for future research in this paradigm.

As a result, diffusion-based approaches are fundamentally redefining medical image segmentation. They position the field not merely as a discriminative recognition task, but as a generative modeling problem. The reviewed works illustrate a clear progression toward controllable and semantically steerable synthesis pipelines. As these methods mature, they will likely converge with prompt-driven foundation models like SAM and CLIP. This convergence is expected to create unified frameworks capable of generating, augmenting, and segmenting medical imagery. Ultimately, such frameworks offer a principled response to the persistent challenges of annotation scarcity and domain shift in clinical deployment.

### 3.6. Multimodal and Hybrid Approaches

In the field of medical imaging, the integration of information from different modalities (e.g., PET, CT, MRI) is of critical importance for capturing both anatomical and functional details. Multimodal and hybrid segmentation methods developed for this purpose have substantially improved the diagnostic accuracy and clinical generalizability of models. In particular, PET/CT fusion has become a fundamental component of automated segmentation systems by enabling the simultaneous examination of the metabolic activity of tumor tissues alongside their anatomical boundaries. For instance, Bi et al. [66] demonstrated that combining PET and CT data through late-fusion architectures yielded a significant performance improvement of up to 15.26% in the Dice coefficient compared to single-modality approaches. Such approaches enable the high-level integration of features derived from different modalities.

Fang et al. [67], achieved more robust performance in brain tumor segmentation by combining multi-sequence MRI data through a self-supervised hybrid fusion network approach. The model offered an architecture capable of automatically balancing contrast differences while simultaneously processing local and global features. Similarly, Karthik et al. [68] developed an ensemble-based system for PET/MRI fusion-based tumor segmentation, achieving a significant advancement in both diagnostic accuracy and interpretability. This model presented a hybrid architecture robust to clinical data heterogeneity and automatically optimized modality-based weighting. In addition, the Multiroimix method proposed by Wang et al. [69] reduced training imbalance and improved the detection of small lesions by combining data augmentation techniques in a multimodal manner on PET/CT datasets. Such approaches indicate the potential for integration in multimodal systems not only at the level of combined input, but also throughout the training process. Finally, the systematic review published by Basu et al. [70] analyzed the effects of CT-MRI-PET fusion on diagnostic segmentation, highlighting that early fusion strategies are limited in terms of data compatibility, whereas hybrid fusion approaches produce more flexible and clinically meaningful outputs.

In summary, multimodal and hybrid approaches represent a critical frontier in medical image segmentation, where the complementary strengths of anatomical, functional, and sequential imaging modalities are leveraged to overcome the inherent limitations of any single data source. The reviewed studies collectively illustrate a methodological evolution from straightforward late-fusion pipelines toward self-supervised, ensemble-based, and augmentation-aware hybrid architectures that adaptively integrate modality-specific information. As multimodal acquisition protocols become increasingly standardized in clinical workflows, future research must prioritize the development of robust fusion frameworks. These systems must be resilient to missing modalities, inter-site acquisition variability, and class imbalance. Ultimately, this will ensure that diagnostic gains observed in controlled benchmarks are reproducible in real-world clinical environments.

### 3.7. Emerging Paradigms (Foundation Models, Promptable Segmentation, Self-Supervised Segmentation)

The paradigm shift observed in medical image segmentation in recent years is characterized by a transition from classical task-specific models toward foundation model-based generalizable artificial intelligence systems. These models are trained on vast volumes of multimodal medical data, learning reusable representations across diverse tasks [71]. The most significant contribution of foundation models lies in their ability to achieve high performance with limited labeled data and to facilitate transfer learning and domain-specific adaptation.

In this paradigm, the promptable segmentation approach integrates user guidance directly into the model’s decision-making process. For instance, the PROMISE (Prompt-driven 3D Medical Image Segmentation) system proposed by Li et al. [72] achieved single-point segmentation in 3D volumes by leveraging pretrained 2D foundation model knowledge. Similarly, AutoProSAM, developed by Li et al. [73], extended the SAM architecture with automatic prompt generation for 3D organ segmentation. These models have translated the “text-to-segmentation” and “prompt-to-mask” paradigms into the medical domain, establishing an interactive learning process driven by expert feedback [74].

Self-supervised learning (SSL) is one of the most critical components for training foundation models. The SSL approach developed by Ouyang et al. [75] improved few-shot segmentation performance by performing pretraining on unlabeled medical images. This method combined contrastive learning and adaptive superpixel strategies to more accurately represent organ or lesion boundaries.

Furthermore, the FDAS (Foundation Model Distillation and Anatomic Structure-Aware Learning) method introduced by Qi et al. [76] enhanced the clinical robustness of self-supervised segmentation by combining foundation model distillation with anatomical structure-aware multi-task learning. This work strengthened the interpretability of the model and reported improvements of 6–10% in Dice metrics across multi-organ scenarios.

Finally, Zhou et al. [13] systematically examined the rise of foundation models in medical image segmentation, designating this new era as the “foundation model era.” The study anticipates that the combination of multimodal data, promptable mechanisms, and self-supervised strategies will constitute the foundation of future medical artificial intelligence architectures.

Taken together, the emerging paradigms reviewed in this section signal a fundamental reconceptualization of medical image segmentation. Models are no longer designed for narrowly defined tasks. Instead, they are pretrained on large-scale, heterogeneous medical data and subsequently adapted through prompts, distillation, or lightweight fine-tuning. The convergence of promptable segmentation, self-supervised pretraining, and foundation model architectures suggests that future systems will be characterized by interactivity, annotation efficiency, and cross-domain transferability rather than task-specific optimization. Realizing this potential, however, will require addressing persistent challenges. These include computational scalability, the need for clinically validated prompt design, and the interpretability of representations learned from massive and diverse training corpora.

## 4. Dataset Landscape and Benchmarking Protocols

### 4.1. Public Datasets Across Imaging Modalities

The comparable and reproducible evaluation of methods developed in the field of medical image segmentation depends largely on the diversity and representational capacity of the public datasets employed. These datasets reflect the physical constraints and clinical variations specific to different imaging modalities, enabling the testing of algorithm performance under real-world conditions.

MRI-based datasets play a dominant role particularly in neurological and cardiac segmentation studies, owing to their high soft tissue contrast. The BraTS (Brain Tumor Segmentation) dataset, one of the most well-established benchmarks in this domain, explicitly reflects heterogeneity and domain shift problems through its multi-center and multi-scanner MRI data. The separate annotation of tumor sub-regions enables the evaluation of multi-class segmentation methods [15,16]. In cardiac applications, the ACDC dataset serves as a reference for modeling temporal and structural variations, as it encompasses ventricular and myocardial segmentation across different cardiac phases [12].

PET and PET/CT datasets represent multimodal scenarios that require functional information to be considered alongside anatomical structures. PET images, which are challenging for segmentation due to their low spatial resolution and high noise levels, are most commonly evaluated in conjunction with CT and are employed for testing multimodal fusion approaches. In this domain, multi-center datasets such as AutoPET and HECKTOR, which focuses on head and neck tumors, have become the primary benchmarks for clinical validation of PET/CT segmentation algorithms [77,78].

Ultrasound (US) and endoscopic images present unique segmentation challenges due to factors such as operator dependency, speckle noise, and variable illumination conditions. The BUSI dataset provides pixel-level annotations of breast lesions, serving as an important reference for US segmentation [79], while endoscopic datasets such as CVC-ClinicDB are widely employed in real-time and video-based segmentation studies [80]. The key public datasets discussed in this section, along with their modalities, target tasks, data sizes, and primary references, are summarized in Table 1.

### 4.2. 2D vs. 3D Datasets and Multi-Organ Considerations

Public datasets differ significantly not only in terms of imaging modality, but also with respect to 2D or 3D representations and single-organ or multi-organ annotations. This distinction directly influences the design of segmentation architectures and evaluation protocols. Two-dimensional datasets offer lower computational cost and greater sample diversity. However, they provide limited representation of volumetric contextual information. In contrast, 3D datasets enable the modeling of anatomical continuity and inter-organ spatial relationships. Yet they present challenges such as higher memory requirements and limited sample sizes. This has heightened the importance of comparative studies examining the performance–efficiency trade-off among 2D, 2.5D, and 3D approaches.

In this context, multi-organ datasets present more challenging benchmarks that test the ability of models to learn not only local structural information but also global anatomical context. The MSD, with its multi-task structure encompassing diverse organ systems and pathologies, has enabled the evaluation of the generalizability of segmentation methods across organs and modalities [82]. Similarly, abdominal multi-organ datasets such as BTCV [83] and AMOS [84] explicitly reflect challenges such as ambiguous organ boundaries and inter-class imbalance.

Such 3D and multi-organ benchmarks have revealed, beyond architectural complexity, the decisive role of preprocessing, resampling, and training protocols on performance. In particular, the consistent performance of the nnU-Net framework across diverse datasets underscores the importance of data-driven and automatic configuration approaches [31].

### 4.3. Annotation Quality, Inter-Rater Variability, and Reproducibility

The reliability of performance results reported in medical image segmentation depends largely on the quality of annotations and the clinical ground truth employed. However, since many public datasets contain labels produced by different clinical experts, inter-rater variability emerges as an unavoidable problem. Particularly in lesions with ambiguous boundaries and low-contrast anatomical structures, disagreements among experts complicate the objective evaluation of segmentation outputs [32].

The impact of annotation ambiguity on evaluation outcomes has been clearly observed in biomedical image analysis challenges. Maier-Hein et al. [35] demonstrated that different evaluation metrics and minor protocol variations can substantially alter the relative ranking of methods on the same dataset. This finding reveals that comparisons based on a single performance metric may be misleading and that segmentation results cannot be interpreted independently of context.

The sensitivity of evaluation metrics to annotation ambiguity is another important factor. Yeghiazaryan and Voiculescu [33] reported that volume-based metrics (e.g., Dice) and boundary-based metrics (e.g., Hausdorff distance) are affected by inter-rater variability to different degrees. In particular, the excessive sensitivity of boundary-based metrics to minor annotation differences may result in clinically insignificant discrepancies being reported as substantial performance variations.

In addition to these issues, differences in data preprocessing steps, data splitting strategies, and metric computation details are among the primary factors limiting the reproducibility of benchmark results. In the literature, large performance discrepancies reported across studies on the same dataset are frequently attributable to differences in experimental protocols rather than architectural innovations [31,35]. For this reason, in modern review and benchmark studies, it is of critical importance to explicitly report not only accuracy values, but also the annotation process, evaluation protocol, and metric selection.

## 5. Comparative Analysis Across Modalities and Tasks

### 5.1. Comparative Trends Across Imaging Modalities

The comparative analyses presented in this section are based exclusively on findings reported in the cited benchmark studies. No new experiments were conducted; all performance observations are traceable to the referenced works and the public datasets listed in Table 1.

The performance of methods in medical image segmentation is strongly dependent not only on the architectural design employed, but also on the physical and clinical characteristics inherent to each imaging modality. MRI, CT, PET, and endoscopic images differ substantially in terms of contrast mechanisms, spatial resolution, noise structure, and artifact profiles, differences that directly determine the conditions under which segmentation algorithms succeed or fail. Understanding these modality-specific dynamics is therefore essential for interpreting benchmark results and for designing systems that generalize reliably across clinical contexts.

MRI-based segmentation offers a relatively advantageous environment for deep learning methods by virtue of its high soft tissue contrast and multi-sequence structure. The BraTS benchmark, employed particularly in brain tumor segmentation, has become a standard reference for performance comparisons across different architectures. Comprehensive analyses conducted on BraTS have demonstrated that the effective integration of multi-sequence information and the training protocol are more decisive than the architectural type itself [15,16]. Furthermore, automatic configuration approaches such as nnU-Net have shown that more consistent and generalizable performance can be achieved on MRI datasets by reducing architectural dependency [31].

CT segmentation presents a distinct challenge profile due to lower soft tissue contrast and ambiguous organ boundaries. The LiTS and KiTS benchmarks, focusing on liver and kidney segmentation, respectively, explicitly reflect problems of inter-class imbalance and intensity similarity [17,81]. Studies conducted on these datasets have demonstrated that 3D approaches capable of capturing broad contextual information provide an advantage; however, performance gains frequently originate from data preprocessing, resampling, and training strategies rather than architectural innovations.

Endoscopic images exhibit a distinct segmentation dynamic due to variable illumination conditions and tissue heterogeneity. Early studies on colon polyp segmentation demonstrated that contextual mapping and attention-based approaches play an important role in identifying small and irregular lesions [80]. In this modality, the requirement for real-time processing renders computational efficiency a critical factor in architectural design.

Overall, segmentation performance is highly sensitive to modality-specific data characteristics, preprocessing, and training protocols. This observation has important methodological implications. Direct comparisons of results across different modalities must be approached with caution. Furthermore, claims of universal superiority for any single architecture remain scientifically unsupported [31,35]. Therefore, rather than pursuing a one-size-fits-all solution, the field requires modality-aware evaluation frameworks. These frameworks must explicitly account for the physical constraints and clinical requirements of each imaging context.

### 5.2. Task-Dependent Performance Characteristics

Regardless of the imaging modality, the performance of methods in medical image segmentation depends largely on the intrinsic nature of the segmentation task itself. Different task types, including the segmentation of small-volume lesions, multi-organ delineation, and the separation of low-contrast anatomical structures, impose fundamentally different demands on model design, loss function selection, and evaluation methodology. Recognizing these task-dependent characteristics is essential for interpreting reported results and for developing architectures that are genuinely fit for clinical purpose.

The segmentation of small and irregular lesions (Small Lesion Segmentation) is one of the most challenging scenarios due to class imbalance and boundary ambiguity. BraTS studies conducted on brain tumor sub-regions have demonstrated that Dice scores for small sub-structures such as the enhancing tumor are consistently lower compared to the tumor core [15,16]. Similarly, in the LiTS benchmark, the segmentation of small liver metastases yields significantly lower performance compared to larger lesions [17]. This highlights the critical importance of multi-scale feature extraction and contextual attention mechanisms for small target segmentation.

Multi-organ segmentation requires the modeling of anatomical context and the precise delineation of inter-class boundaries. The MSD, with its structure encompassing diverse anatomical regions and pathologies, has provided an important benchmark for testing the generalizability of multi-task and multi-organ segmentation [82]. Studies conducted on such datasets have demonstrated that model performance is strongly dependent not only on architectural depth, but also on preprocessing and training strategies. In particular, the consistent performance of nnU-Net across a large number of tasks underscores the importance of data-driven and automatic configuration approaches [31].

In 3D segmentation tasks, inter-slice consistency and volumetric continuity are of critical importance. Three-dimensional benchmarks such as LiTS and KiTS have demonstrated that 2D approaches can produce inter-slice inconsistencies, and that 3D contextual modeling strategies yield more reliable results particularly in clinical volume estimation [17,81]. Nevertheless, the nnU-Net study has shown that 2D, 3D, and hybrid configurations exhibit task-dependent performance profiles that vary across different tasks [31].

When evaluated overall, a clear trend emerges from the literature. Segmentation performance is determined more by task type, target size, and class imbalance than by the choice of architectural family. This has important implications for method validation. Mean Dice scores alone are insufficient to characterize performance without an explicit task context. Consequently, future benchmarks should adopt task-stratified evaluation protocols. Reporting performance separately across different lesion sizes and dimensionality configurations will enable more meaningful clinical comparisons.

### 5.3. Key Performance Trade-Offs: Accuracy, Robustness, and Efficiency

The evaluation of methods in medical image segmentation cannot be conducted solely on the basis of accuracy metrics. Modern literature demonstrates that segmentation performance must be assessed along three fundamental dimensions: accuracy, robustness/generalizability, and computational efficiency. The balance among these three dimensions is of critical importance, particularly in systems intended for clinical application.

The most widely used metrics in segmentation studies are the Dice similarity coefficient and Hausdorff distance. However, it has been emphasized in the literature that high Dice scores do not always correspond to clinically meaningful outcomes [32]. For instance, in the BraTS benchmark, although some methods produce similar mean Dice values, notable performance differences in small tumor sub-regions have been reported [16]. Similarly, in the LiTS and KiTS datasets, significant discrepancies between volumetric accuracy and boundary precision have been observed [17,81]. This demonstrates that accuracy alone is not a sufficient indicator of performance.

A model achieving high accuracy does not necessarily imply that it will demonstrate equivalent performance on data acquired from different centers or different scanners. In a systematic analysis of biomedical image analysis challenges, it has been shown that even minor changes in data splitting can affect method rankings [35]. Furthermore, the nnU-Net study demonstrated that data-driven configuration strategies yield more robust results across diverse tasks compared to architectural innovation [31]. These findings indicate that robustness is closely related not only to network depth, but also to training protocols and data processing pipelines.

Computational cost and memory requirements constitute significant constraints for segmentation systems intended for use in clinical settings. Despite the advantage of 3D networks in modeling volumetric context, their high GPU memory demands can be limiting in practical applications. The ability of nnU-Net to automatically select between 2D and 3D configurations for different tasks has demonstrated that the accuracy-efficiency trade-off is task-dependent [31]. Similarly, in endoscopic segmentation studies, it has been reported that real-time processing requirements constrain model complexity and that lightweight architectures are preferred [80].

## 6. Decision Framework: When to Choose Which Method?

### 6.1. Key Factors Influencing Method Selection

The selection of an appropriate method in medical image segmentation cannot be made solely on the basis of the highest accuracy values reported in the literature. As discussed in the preceding sections, performance results are strongly dependent on modality, task type, and data characteristics. Model selection must therefore be approached as a multidimensional decision-making process.

The volume of the dataset and annotation quality are the primary factors determining model complexity. In large and heterogeneous datasets, data-driven automatic configuration approaches can achieve high generalizability. The consistent performance of nnU-Net across diverse tasks has demonstrated the importance of data-compatible configuration over architectural innovation [31]. In contrast, in scenarios with limited data, the tendency of over-parametric models toward overfitting has been frequently reported in the literature [14].

Different imaging modalities such as MRI, CT, and PET play a decisive role in model selection. While multi-sequence integration is of critical importance in MRI [15], intensity homogeneity and class imbalance may be more determining factors in CT segmentation [17]. In PET segmentation, multimodal fusion approaches have produced more stable results compared to unimodal models [66]. Model selection must therefore be made with due consideration of the physical constraints inherent to the modality.

In endoscopic or intraoperative applications requiring real-time processing, lightweight and computationally efficient architectures are preferred [80]. Despite the high accuracy potential of 3D deep networks, memory requirements and latency are significant limiting factors for clinical integration. Method selection must therefore be made not solely on the basis of accuracy, but with careful consideration of the full deployment context, including hardware constraints, acceptable inference latency, integration requirements within existing clinical workflows, and the regulatory standards governing the use of AI-assisted tools in medical practice. A method that achieves state-of-the-art performance on a benchmark dataset but demands prohibitive computational resources or fails to meet real-time processing thresholds offers limited translational value, regardless of its theoretical merits.

### 6.2. Strengths and Limitations of Major Segmentation Paradigms

CNN-based encoder-decoder architectures have long been the dominant approach in medical image segmentation. The U-Net architecture in particular has become the primary reference model in the field, owing to its ability to achieve effective performance even on biomedical datasets with limited annotations and to integrate low-level spatial information with high-level contextual representations [2]. While this architecture offers advantages such as relatively low computational cost and ease of training, its limited capacity to model volumetric context when applied in a slice-based (2D) manner emerges as a significant limitation. Indeed, early analyses conducted on the BraTS benchmark demonstrated that contextual insufficiency in small tumor sub-regions can reduce performance [15,16]. In datasets such as LiTS and KiTS, where small metastatic lesions occupy a disproportionately small pixel fraction, CNN-based models consistently underperform on minority structures even when mean Dice scores remain high.

Transformer-based models such as TransUNet and Swin-UNet distinguish themselves through their capacity to learn global context and long-range dependencies, surpassing the limited local receptive field of CNNs [5]. However, the most significant limitations of these models are their high computational cost and their requirement for very large datasets. When trained on limited data, they are prone to overfitting. For this reason, hybrid architectures that combine the local detail extraction capability of CNNs with the global perception of Transformers generally yield the most consistent results in the literature [6].

Foundation models such as SAM substantially reduce the burden of manual annotation through their zero-shot capabilities and their ability to perform interactive segmentation based on user-provided prompts [8]. Their most notable strength lies in their generic flexibility. However, the fact that these models are trained on natural images leads to performance degradation in low-contrast modalities such as ultrasound, at complex tumor boundaries, and in tasks requiring volumetric (3D) spatial consistency [7]. Although these limitations are partially mitigated by domain-specific fine-tuned variants such as MedSAM, their continued dependence on human intervention in clinical workflows requiring full automation remains a practical constraint [10].

Diffusion-based approaches (e.g., Diffusion U-Net) distinguish themselves through their capacity to generate highly diverse synthetic data and their training stability. They offer superior generative capabilities particularly in rare pathologies with data scarcity or in cases with ambiguous mask boundaries [60]. Nevertheless, the multi-step denoising process considerably increases inference time and computational burden. This currently renders diffusion models impractical for clinical scenarios requiring real-time segmentation, such as robotic surgery or endoscopy.

Taken together, each of the major segmentation paradigms reviewed here presents a distinct trade-off profile: CNN-based architectures offer practical efficiency and annotation robustness but are constrained in global context modeling; Transformer-based models excel at capturing long-range dependencies but demand substantial data and computational resources; foundation models provide remarkable flexibility and interactivity but require domain-specific adaptation to reach their full potential in medical contexts; and diffusion-based approaches enable high-fidelity generative augmentation but remain limited by inference latency. Rather than identifying a universally superior paradigm, these observations suggest that method selection should be driven by a careful alignment between the strengths of a given architecture and the specific demands of the target clinical task, dataset characteristics, and deployment constraints. A structured comparison of the strengths, limitations, ideal use cases, and known failure scenarios of these major segmentation paradigms is provided in Table 2.

### 6.3. Practical Guidelines and Common Failure Scenarios

The practical selection of segmentation methods should not be based solely on theoretical performance comparisons, but should also take into account failure patterns observed under real-world conditions. Many high accuracy values reported in the literature are obtained in controlled benchmark environments, yet fail to demonstrate the same consistency on heterogeneous data with clinical distributions [35]. For this reason, in the model selection process, the differences between the data distribution and the benchmark distribution must be explicitly evaluated.

One of the most frequently encountered failure scenarios in practical applications is the domain shift effect. Images acquired at different centers may exhibit significant variations in terms of scanner manufacturer, acquisition protocol, and reconstruction parameters. Even in multi-center datasets such as BraTS, it has been reported that minor differences between training and test distributions have an impact on performance [16]. Although the nnU-Net study demonstrated that data-driven configuration provides partial robustness against such distributional shifts, full generalizability cannot be guaranteed [31].

Another common failure scenario is the systematic neglect of small and rare classes. In the LiTS benchmark, the segmentation of small metastases can exhibit notable error rates even when mean Dice values are high [17]. Similarly, the omission of small tumor foci in the KiTS dataset can lead to clinically critical consequence [81]. This demonstrates that average performance metrics may mask rare but clinically significant structures.

Boundary ambiguity and annotation variability are also a significant source of model failures. The sensitivity of evaluation metrics to annotation errors is particularly pronounced in boundary-based measures such as Hausdorff distance [32]. Maier-Hein et al. [35], demonstrated that minor metric variations can affect method rankings, emphasizing that interpreting benchmark results as absolute ground truth carries considerable risk.

From a practical guidance perspective, several key principles should be considered when selecting a segmentation model. First, dataset size and annotation density are fundamental determinants: if the dataset is small and volumetric consistency is not critical, lighter 2D architectures may be preferred to reduce the risk of overfitting, whereas if volumetric context is important and sufficient computational resources are available, 3D approaches may provide a meaningful advantage in capturing anatomical continuity. Second, if data heterogeneity is high (for instance in multi-center or multi-scanner settings) data-driven automatic configuration systems such as nnU-Net may produce more balanced and reproducible results by adapting preprocessing and training strategies to the specific characteristics of the data [31]. Third, the nature of the target structure must be considered: small and rare lesions demand loss functions and sampling strategies specifically designed to counteract class imbalance, while multi-organ tasks require architectures capable of modeling both local boundary precision and global anatomical context simultaneously. Fourth, the deployment environment imposes its own constraints: real-time applications such as endoscopy or intraoperative guidance favor lightweight, low-latency architectures, whereas offline diagnostic workflows may tolerate higher computational costs in exchange for improved accuracy. Finally, the choice of evaluation protocol is as consequential as the choice of architecture, models should be validated not only on standard benchmark splits but also on locally representative data that reflects the specific patient population, scanner characteristics, and clinical workflow of the intended application.

To support practitioners in navigating the decision framework presented in Figure 3, the following step-by-step guidance is offered. The first consideration is dataset size and annotation density: if the dataset is small or annotations are limited, lightweight and data-efficient architectures are preferred, as over-parametric models are prone to overfitting under these conditions. The second consideration is task type and modality: for 3D volumetric tasks where anatomical continuity is critical, Transformer-based architectures offer an advantage, whereas real-time applications such as endoscopic segmentation favor lightweight CNN-based models due to their low inference latency. The third consideration is domain shift: in multi-center or multi-scanner settings, data-driven automatic configuration systems provide more robust and reproducible results, while zero-shot generalization requirements favor SAM-based models. Finally, hardware and deployment constraints must be evaluated, as large foundation models may be prohibitive on standard clinical hardware without parameter-efficient fine-tuning strategies.

To illustrate this process, three brief scenarios are considered. In a single-center brain tumor MRI study with limited annotations, the framework directs toward lightweight CNN-based automatic configuration, which adapts to dataset characteristics and has demonstrated consistent performance on brain tumor segmentation tasks. In a multi-center abdominal CT study with high domain shift, Transformer-based architectures combined with data-driven preprocessing provide a more robust solution. In a real-time colonoscopy setting, lightweight CNN-based architectures remain the preferred choice given the strict latency requirements. These scenarios demonstrate that the framework is not prescriptive but condition-driven, and that its reliability stems from its grounding in empirical benchmark evidence rather than theoretical architectural preference.

In conclusion, the evaluation of segmentation methods should be conducted not merely through average accuracy values, but through the systematic analysis of failure patterns across diverse conditions. Unless clinical applicability, generalizability, and computational requirements are considered jointly, performance improvements reported in the literature may not translate directly into real-world practice. A rigorous model selection process (one that accounts for task characteristics, data distribution, deployment constraints, and validation methodology) is therefore an indispensable prerequisite for the responsible integration of deep learning-based segmentation into clinical systems.

## 7. Robustness, Generalization, and Clinical Reliability

### 7.1. Robustness to Domain Shift and Acquisition Variability

The reliable deployment of medical image segmentation systems in clinical environments depends on their ability to maintain stable performance across varying data distributions. Images acquired in clinical practice exhibit heterogeneity with respect to numerous factors including scanner manufacturer, magnetic field strength, reconstruction algorithm, and patient population. This gives rise to the domain shift problem, which stems from the discrepancy between the training data distribution and the deployment environment, and can cause dramatic performance degradation in CNNs [14,45].

Although multi-center benchmark datasets partially reflect this heterogeneity, model performance has been reported to be highly sensitive to inter-center distributional shifts. BraTS analyses have demonstrated notable performance variations across data from different institutions [16]. Similarly, performance variations attributable to scanning protocol and patient population have been observed in the LiTS and KiTS datasets [17,81]. Although automatic configuration systems adapted to data characteristics, such as nnU-Net, provide a strong foundation against such shifts, full generalizability cannot be guaranteed [31]. These findings demonstrate that high mean Dice scores do not automatically confer robustness in clinical settings.

One of the most prominent solutions in the literature against the domain shift problem is Unsupervised Domain Adaptation (UDA). UDA aims to transfer knowledge acquired from labeled source data to unlabeled target clinical data. GAN-based bidirectional style transfer models (CycleGAN) can harmonize contrast differences across modalities and scanners without disrupting semantic structure [14]. Additionally, methods such as Fourier Domain Adaptation (FDA), which operates in the frequency domain, achieves rapid domain alignment by modifying only the low-frequency components (global illumination and contrast) of source and target images while preserving high-frequency structural details [85].

Another critical line of defense against variations encountered in clinical settings is provided by Test-Time Augmentation (TTA) and Test-Time Adaptation approaches, which strengthen the model during the inference phase. TTA, applied without the need to retrain the model, generates multiple predictions by applying various transformations to the test image and subsequently aggregates these predictions. Recently, Nazzal et al. [59] applied TTA based on random circular shifts to MedSAM, the medical variant of SAM, substantially improving the model’s boundary sensitivity against minor misalignment errors occurring during clinical acquisition. On the other hand, the test-time adaptation approach proposed by Karani et al. [45] dynamically updates the normalization layers of the segmentation network with respect to the current test image using a denoising autoencoder, thereby offering superior clinical robustness against scanner and protocol variations.

The clinical significance of domain shift becomes particularly evident when segmentation models are deployed across different institutional workflows. In radiotherapy planning, for instance, a model trained on CT data from one scanner manufacturer may produce systematically different organ-at-risk contours when applied to data acquired at a different center, directly affecting dose calculation and treatment safety. Similarly, in oncological follow-up studies conducted across multiple institutions, domain-induced performance variability can compromise the reproducibility of tumor volume measurements required by standardized response evaluation protocols such as RECIST. These examples illustrate that domain shift is not merely a technical benchmarking concern but a clinically consequential phenomenon that must be explicitly addressed before segmentation models can be safely integrated into routine diagnostic and therapeutic workflows.

In essence, genuine clinical robustness extends beyond architectural depth, necessitating the alignment of data distributions through UDA strategies and the integration of inference-phase optimizations such as TTA into the segmentation pipeline as a standard procedure.

### 7.2. Evaluation Sensitivity, Annotation Variability, and Reliability

The clinical reliability of segmentation models is directly related to the characteristic properties of evaluation metrics and annotation quality. The literature demonstrates that evaluations based on a single metric can be clinically misleading [32]. For instance, the Dice coefficient (the most widely used overlap metric) does not account for the spatial distribution of errors; a slight overflow at an anatomical boundary and an isolated false positive prediction in an entirely different region may cause an equal degree of reduction in the Dice score [33]. Although boundary-based measures such as Hausdorff distance (HD95) or ASSD are employed to overcome these blind spots, these metrics may also exhibit excessive sensitivity to minor annotation differences and outliers.

Another fundamental factor influencing evaluation sensitivity is inter-rater variability. Labels accepted as ground truth in medical images are typically based on the subjective assessments of experts. Analyses conducted on comprehensive datasets such as BraTS and ACDC have demonstrated that the performance of the most successful deep learning models approaches the boundaries of inter-rater variability (human-level performance). Once this threshold is surpassed, marginal metric improvements may indicate not that the model is clinically superior, but merely that it has overfit to the labeling style (bias) of a particular expert.

The impact of annotation ambiguity on evaluation outcomes has also been demonstrated in the large-scale benchmark analyses conducted by Maier-Hein et al. [35]. This study revealed that minor metric choices or small variations in test data can dramatically alter the success rankings of algorithms. Particularly in the segmentation of small lesions in datasets such as LiTS and KiTS, average performance metrics may mask rare but clinically critical errors, such as the complete omission of a small tumor focus [17,81].

Finally, uncertainty quantification is increasingly becoming a standard requirement in modern systems to overcome these evaluation limitations and ensure clinical reliability. Techniques such as Monte Carlo Dropout or Bayesian neural networks enable the model to produce not only a segmentation mask, but also a pixel-level confidence map. This allows ambiguous boundaries or suspicious lesions where the model remains uncertain to be presented for clinician review. In this context, clinical reliability should be supported not solely by high Dice scores, but by the transparent reporting of metric selection, the explicit acknowledgment of expert variability, and the model’s ability to express its own uncertainty. A structured comparison of the focus criteria, strengths, and clinical blind spots of the evaluation metrics discussed in this section is provided in Table 3.

Beyond uncertainty quantification, algorithmic fairness represents an increasingly critical dimension of clinical reliability. Segmentation models trained on datasets that underrepresent certain patient populations, such as specific age groups, ethnicities, or disease subtypes, may exhibit systematically degraded performance on underrepresented subgroups, introducing a form of clinical inequity that aggregate metrics such as mean Dice scores fail to reveal. Addressing fairness in medical image segmentation therefore requires not only diverse and representative training data, but also subgroup-stratified evaluation protocols that explicitly assess performance disparities across clinically relevant patient categories.

### 7.3. Toward Clinically Reliable Deployment

The clinical integration of segmentation systems requires, above all, reproducibility and methodological transparency. It has been demonstrated that results reported across different studies on the same dataset can be substantially influenced by differences in experimental protocols (e.g., preprocessing steps, data splitting strategies) rather than architectural innovations [31]. For this reason, training and validation configurations, hyperparameter choices, and external validation procedures must be explicitly reported in the transition to clinical use.

From a clinical utility perspective, segmentation outputs must be evaluated not solely through pixel-level metrics, but also through their impact on volumetric consistency and clinical decision support processes (treatment planning, tumor burden estimation). Large-scale 3D datasets such as LiTS and KiTS clearly reflect the indirect importance of precise organ or lesion boundary delineation (and consequently the accuracy of volumetric measurements) in clinical diagnosis and radiotherapy planning [17,81].

One of the most significant practical barriers to reliable clinical deployment is data privacy constraints. In this regard, Federated Learning offers a critical solution for clinical integration by enabling the training of multi-center models without transferring patient data outside the institution [86,87]. Furthermore, federated benchmarking platforms across institutions are becoming an indispensable component of modern medical artificial intelligence workflows, enabling the safe and reliable assessment of the real-world performance and generalizability of developed algorithms [88].

Finally, establishing clinical reliability and making artificial intelligence deployable in the field requires not the imposition of full automation, but rather Human-in-the-Loop and interactive segmentation approaches that integrate the expert into the process [89,90]. Systems in which clinicians can rapidly correct model outputs through user-friendly prompts and are directed toward suspicious regions via uncertainty maps generated by the model enhance clinical trust in artificial intelligence, render legal liabilities manageable, and create a more sustainable framework for the responsible adoption of AI-assisted tools in routine clinical practice [91].

The challenges of real-world deployment are further illustrated by considering two representative clinical workflow scenarios. In a multi-center lung cancer screening program, a segmentation model developed and validated at a single institution may encounter substantial performance degradation when deployed across partner sites with different CT scanner models, reconstruction kernels, and patient demographics. Without prospective cross-site validation and domain adaptation strategies, such degradation can go undetected until it produces clinically consequential errors in nodule volume estimation or lesion boundary delineation. In a surgical planning workflow for liver resection, preoperative 3D segmentation of the liver and surrounding vasculature must meet strict accuracy and reproducibility standards, as errors in organ boundary delineation directly influence the surgical approach and patient safety. These scenarios underscore that cross-dataset generalization is not an abstract benchmark metric but a concrete clinical requirement, and that the responsible deployment of segmentation models demands prospective validation under conditions that closely reflect the intended clinical environment.

In conclusion, clinical reliability is a multidimensional requirement that extends well beyond architectural performance. It necessitates methodological transparency in the reporting of experimental protocols and evaluation procedures, multi-center validation frameworks that preserve data privacy through federated learning, and interactive system designs that place the clinician at the center of the decision-making process. No segmentation model can be considered clinically reliable based solely on benchmark performance. It must be externally validated on locally representative data and evaluated across metrics that capture both volumetric accuracy and boundary fidelity. Ultimately, these models should be integrated into workflows that support, rather than replace, clinical judgment. Achieving this standard requires a concerted effort across algorithm developers, clinical practitioners, and regulatory bodies to establish shared benchmarking protocols, uncertainty-aware evaluation frameworks, and human-centered deployment pipelines that are robust, interpretable, and accountable in real-world medical practice.

## 8. Open Challenges and Future Research Directions

### 8.1. Standardization of Benchmarks and Reporting

One of the most fundamental problems that makes the clinically meaningful interpretation of comparative studies in medical image segmentation difficult is the heterogeneity across datasets, evaluation protocols, and metric selections. Comprehensive benchmark analyses have demonstrated that minor changes in evaluation metrics, ranking computation methods, or strategies for handling missing data can dramatically alter the success rankings of algorithms. For instance, a large-scale investigation conducted by Maier-Hein et al. compellingly demonstrated that the removal of a single image from a test dataset, or even minor changes in metric variants, can alter the winner of biomedical imaging challenges. This necessitates that performance improvements reported in the literature be interpreted contextually in relation to the protocol employed, rather than as evidence of absolute superiority [35].

Another critical gap in benchmarking processes is the lack of methodological reporting. In the vast majority of biomedical image analysis challenges, key experimental details are not reported with sufficient transparency. These include the experience level of experts performing ground truth annotation, hyperparameter selections, and data augmentation strategies, all of which directly influence results. To address this deficiency and ensure the reproducibility of challenge results, comprehensive reporting guidelines such as BIAS (Transparent Reporting of Biomedical Image Analysis Challenges) have been developed by Maier-Hein, et al. [92]. Similarly, the standardization of guidelines such as CONSORT-AI [93] and SPIRIT-AI [94], which mandate methodological transparency for the integration of artificial intelligence interventions into clinical studies, represents a critical step toward clinical reliability.

Another fundamental component of future standardization is the testing of models for general validity and robustness rather than performance on a single specific task. In this context, multi-task evaluation frameworks such as MSD [95] and multi-organ/multi-modality (CT-MR) focused benchmarks such as the CHAOS Challenge [96] have constituted the most important milestones in the field for fairly comparing the generalizability of methods across different anatomical regions.

Finally, there is a need for evaluation systems that move beyond conventional pixel-based metrics and place clinical utility at the center, as well as for architectures that overcome data privacy constraints. Federated benchmarking platforms and federated semi-supervised learning approaches [88,97], which enable the safe measurement of algorithms’ real-world performance without transferring patient data outside the institution, will be among the future gold standards of medical artificial intelligence workflows.

### 8.2. Data Efficiency and Scalability

Deep learning-based segmentation models typically require enormously large labeled datasets to achieve high performance. However, in the field of medical imaging, since precise pixel- or voxel-level annotation requires high clinical expertise, constructing large-scale, high-quality datasets is extremely costly and time-consuming [14,41]. Although data-driven automatic configuration systems such as nnU-Net have demonstrated the ability to produce strong results across diverse tasks by extracting maximum utility from available limited data [31], data scarcity remains one of the most significant bottlenecks in the field [14].

To overcome this obstacle and enhance data efficiency, future research must orient toward strategies capable of leveraging vast unlabeled clinical data. Semi-supervised and self-supervised learning approaches are particularly prominent in this regard. For instance, the self-supervised framework developed by Ouyang et al. [75] demonstrated that by performing pretraining on unlabeled medical images through contrastive learning, the model can achieve high segmentation performance even with very few labeled examples (few-shot learning).

On the other hand, scalability refers not only to the growth of datasets, but also to how the increasingly growing model complexity can be managed within hardware constraints. Foundation models trained on massive data such as SAM [8] and their domain-specific medical adaptations such as MedSAM [10] offer remarkable flexibility and generalizability on previously unseen (zero-shot) data. However, the training and deployment of these models with billions of parameters on standard clinical hardware (local servers or edge devices) creates a significant computational bottleneck [7,71].

To address this scalability challenge, Parameter-Efficient Fine-Tuning (PEFT) techniques have become indispensable in modern systems. Rather than retraining the entire model, approaches such as Medical SAM Adapter [97] insert small adapter modules into the network, while techniques such as LoRA (Low-Rank Adaptation) [98] update only a very small subset of parameters, thereby dramatically reducing computational and memory costs and enabling the clinical scalability of large-scale models.

In conclusion, future research directions in medical image segmentation will converge on three interconnected frontiers: generative artificial intelligence applications that synthetically enrich limited training data to mitigate annotation scarcity; self-supervised and semi-supervised algorithms that minimize the dependence on human labeling by extracting meaningful representations from vast unlabeled clinical archives; and parameter-efficient fine-tuning strategies that bridge the gap between the extraordinary capacity of large-scale foundation models and the computational realities of clinical deployment. In parallel, paradigm-specific research priorities have emerged for the three most rapidly evolving model families reviewed in this survey. For VLM-based approaches, the development of lightweight, domain-adaptive architectures capable of achieving reliable pixel-level alignment with clinical terminology under limited data and compute conditions represents a critical unmet need. For SAM-based models, future work must prioritize automatic prompt generation strategies that eliminate user-dependent variability, as well as volumetric extensions that ensure anatomical continuity in 3D segmentation tasks. For diffusion-based approaches, accelerated sampling strategies and hybrid discriminative-generative architectures are needed to reduce inference latency to clinically acceptable levels while preserving the generative fidelity that distinguishes these models from classical segmentation networks. Progress along these interconnected frontiers will not only advance segmentation accuracy, but will also determine whether the remarkable benchmark gains of the past decade can be translated into robust, scalable, and equitable tools that are genuinely useful across the full diversity of real-world clinical environments. A comparative overview of the parameter counts, computational loads, and performance characteristics of representative SOTA models discussed in this section is provided in Table 4.

An additional frontier that merits dedicated attention is the intersection of federated learning and foundation models. While federated learning has been established as a privacy-preserving framework for multi-center model training, its combination with large-scale foundation models such as SAM and VLMs introduces new challenges related to communication efficiency, heterogeneous data distributions across institutions, and the computational feasibility of fine-tuning billion-parameter models in a federated setting. The development of parameter-efficient federated fine-tuning strategies, building on approaches such as LoRA and adapter-based methods, represents a promising direction toward foundation models that are simultaneously generalizable, privacy-preserving, and clinically deployable across diverse institutional environments.

### 8.3. Reproducibility and Transparent Reporting

As the complexity of deep learning algorithms in the segmentation literature increases, the reproducibility of obtained results by independent researchers is becoming an increasingly critical issue. For medical artificial intelligence studies to be considered reliable, a model is expected to produce consistent results when retrained with the same dataset and training protocol; however, the fact that datasets, source code, and trained models are generally not shared publicly makes independent verification of results and the advancement of the field considerably more difficult [99]. Insufficient reporting of data preprocessing steps, data splitting strategies, hyperparameter optimizations, and hardware constraints in deep learning workflows impedes the re-implementation of developed methods in real-world scenarios and their fair comparison.

The extent of this methodological transparency deficit was compellingly demonstrated in a comprehensive study conducted by Maier-Hein et al. [35] across 150 biomedical image analysis challenges. The study revealed that critical information directly influencing algorithm rankings remained undisclosed in the vast majority of tasks. Specifically, how missing data were handled was not reported in 82% of the studies. Furthermore, the use of external training data and the expert annotation process were not reported in 85% and 66% of the studies, respectively. This level of ambiguity renders the independent verification of performance improvements reported in the literature and the fair comparison of different methods virtually impossible.

For future research to overcome this bottleneck, the adoption of open science and full transparency policies is essential. Sharing algorithms as open-source in code repositories must become a fundamental standard. Moreover, to prevent deviations arising from differences in library versions or hardware (GPU) configurations, encapsulating the training and inference environments of models using container technologies such as Docker, as recommended in the KiTS19 benchmark by Heller et al. [81], represents a robust solution strategy. The consistent success achieved by end-to-end automatic frameworks such as nnU-Net, which explicitly presents its entire hyperparameter configuration, demonstrates the catalytic role of methodological transparency in the advancement of the field [31].

Finally, in segmentation studies aimed at clinical integration, strict adherence to international reporting guidelines such as CONSORT-AI [93] and SPIRIT-AI [94], which render the reliability and auditability of artificial intelligence interventions verifiable by independent parties, is not merely a procedural formality, but a scientific and ethical imperative. Open code sharing, complete and granular documentation of experimental details, standardized reporting conventions, and the public release of pretrained models collectively form the foundation upon which meaningful scientific progress can be built and independently verified. Without these safeguards, even the most technically sophisticated segmentation models risk remaining isolated laboratory achievements whose real-world validity cannot be established. Embracing open science principles is therefore not only a prerequisite for reproducibility, but a necessary condition for the responsible translation of deep learning-based segmentation into clinically reliable, equitable, and trustworthy decision support systems.

## 9. Conclusions

This review has comprehensively examined methods in the field of medical image segmentation from the perspectives of the dataset ecosystem, task types, methodological paradigms, and clinical applicability. The literature analysis demonstrates that segmentation performance cannot be explained solely by architectural innovations; data characteristics, task context, evaluation protocols, and the deployment environment play a decisive role in determining performance. In particular, established benchmarks such as BraTS, LiTS, KiTS, and MSD have systematically revealed the task-specific strengths and weaknesses of methods, while also demonstrating that metric selection and protocol differences can substantially influence the interpretation of results [31,35].

This evolution, extending from classical task-specific U-Net and its variants, through the global context capability of Transformers, to prompt-driven foundation models such as SAM, and further to diffusion-based generative approaches, clearly demonstrates that the field is progressing toward more flexible, multimodal, and interactive systems [7,8,10]. However, as the decision support framework presented in our study also emphasizes, no algorithm is universally optimal for all clinical scenarios. Given practical challenges such as clinical objectives, hardware constraints, data size, and domain shift, a context-aware analytical balance must always be maintained between large high-parameter models and computationally efficient conventional or hybrid models [14].

In conclusion, the future of medical image segmentation lies not merely in improving mathematical overlap metrics (such as the Dice score), but in ensuring that models are generalizable to unseen clinical data (robustness), capable of expressing their own uncertainty through uncertainty maps (uncertainty quantification), and designed as interactive “human-in-the-loop” systems that integrate the expert clinician into the decision-making process [32]. Developing algorithms in accordance with open data and code-sharing policies is crucial. Furthermore, presenting them in compliance with international reporting standards (e.g., CONSORT-AI, SPIRIT-AI) will ensure that these extraordinary technical achievements transcend laboratory boundaries and become reliable components of daily clinical practice.

## Figures and Tables

**Table 1:** Common Benchmark Datasets for Medical Image Segmentation.

Dataset	Modality	Target Anatomical Region/Task	Data Size	Study
BraTS ^1^	MRI (T1, T1c, T2, FLAIR)	Brain Tumor (WT, TC, ET) Sub-regions.	~2000 + 3D Volumes	[15,16]
LiTS ^2^	CT Portal Venous Phase)	Liver and Liver Tumor.	201 3D Volumes	[17]
KiTS ^3^	CT	Kidney and Kidney Tumor.	~300 3D Volumes	[81]
MSD ^4^	MRI, CT	10 Different Tasks (Heart, Pancreas, Spleen, etc.).	~2633 3D Volumes	[82]
ISIC ^5^	Dermoscopy	Skin Lesions/Melanoma.	Thousands of 2D Images	[23]
ACDC ^6^	Cine MRI	Cardiac (Right/Left Ventricle, Myocardium).	150 Patients (3D Time Series)	[12]

^1^ https://www.med.upenn.edu/cbica/brats/ (accessed on 1 May 2026). ^2^ https://competitions.codalab.org/competitions/17094 (accessed on 1 May 2026). ^3^ https://kits19.grand-challenge.org/ (accessed on 1 May 2026). ^4^ http://medicaldecathlon.com/(accessed on 1 May 2026). ^5^ https://www.isic-archive.com/ (accessed on 1 May 2026). ^6^ https://www.creatis.insa-lyon.fr/Challenge/acdc/ (accessed on 1 May 2026).

**Table 2:** Comparison of Basic Segmentation Paradigms.

Paradigm	Ref.	Advantages	Disadvantages	Ideal Use Case	Known Failure Scenarios	Annotation Requirements
CNNs (Classical and Automatic)	U-Net [2]; nnU-Net [31]	High efficiency, stable training even with limited data, low inference time.	Inability to capture long-range dependencies (global context).	Standard hardware, homogeneous contrast, and real-time tasks.	Reduced performance in small tumor sub-regions due to contextual insufficiency; inter-slice inconsistency in 3D volumetric tasks.	Full pixel-level annotation required; performance degrades significantly with limited labeled data.
Transformers & Hybrid	TransUNet [5]; Swin-UNet [4]	Superior modeling of complex organ boundaries and global context via self-attention.	Very high hardware and data requirements, risk of losing local details.	Large datasets, complex 3D tumor and multi-organ segmentation.	Prone to overfitting when trained on limited medical datasets; risk of losing local boundary detail in fine-grained structures.	Large volumes of fully annotated data required; particularly sensitive to annotation scarcity due to high parameter count.
Foundation Models (SAM)	SAM [8]; MedSAM [10]	High generalizability, interactive segmentation, reduced annotation burden.	Domain shift in medical images, 3D volumetric constraints, high hardware cost.	Interactive clinical systems (human-in-the-loop) and out-of-domain validation (zero-shot).	Performance degradation in low-contrast modalities such as ultrasound and at complex tumor boundaries; inconsistent 3D volumetric spatial consistency; continued dependence on human prompts limits full automation.	Minimal annotation required; interactive prompts (points, bounding boxes) replace dense pixel-level labels, enabling low-annotation deployment.
Diffusion Models	Diffusion U-Net [60]	Exceptionally high fidelity, uncertainty estimation, synthetic data generation.	Very slow inference time, high computational burden, optimization difficulty.	Segmentation of rare pathologies with ambiguous boundaries and data scarcity.	Clinically impractical inference latency for real-time scenarios such as robotic surgery or endoscopy.	Moderate annotation required for supervised segmentation; generative capabilities partially compensate for data scarcity through synthetic augmentation.
VLMs	PMC-CLIP [54] LLaVA-Med [55]	Semantic guidance via natural language (prompt), high multimodal awareness, and zero-shot flexibility.	Alignment challenges at the pixel level for medical terms, high pretraining cost on clinical data.	Text-guided segmentation and diagnostic systems integrated with radiology reports.	Pixel-level misalignment for anatomically ambiguous medical terms; limited performance without domain-specific pretraining on clinical data.	Text-image pair annotations required; pixel-level masks not always necessary, but domain-specific text-image alignment data is needed for clinical adaptation.

**Table 3:** Comparison and Sensitivity of Evaluation Metrics.

Evaluation Metric	Focus Criterion	Strengths	Clinical Weaknesses/Blind Spots	Reference
Dice Similarity Coefficient (DSC)	Volumetric Overlap	Widely accepted, effective on imbalanced datasets.	Ignores the spatial distribution of errors; disproportionately penalizes small spurious regions in unrelated areas.	[33,34]
Intersection over Union (IoU)	Intersection/Union	Similar to Dice but more sensitive to false positives (FP).	Does not adequately reflect shape and contour fidelity (boundary conformity).	[32]
Hausdorff Distance (HD95)	Surface/Boundary Distance	Excellently measures boundary and shape accuracy.	Excessively sensitive to a single erroneous voxel (outlier) in distant regions (mitigated by HD95).	[35]
ASSD	Average Surface Distance	More robust to outliers compared to HD95.	May partially lose sensitivity for very small lesions in terms of size.	[33]

**Table 4:** Parameter, Complexity, and Performance Analysis of SOTA Models.

Method/ Architecture	Parameter Count (M)	Computational Load (FLOPs)	Architectural Basis	Typical Observation/ Performance Inference
U-Net/U-Net++	~9.16 M–31.18 M	~497 G–680 G	Pure CNN	Low-to-moderate complexity, high speed (FPS), baseline reference.
nnU-Net	~34.29 M–150 M	~554 G	Automatic Configuration	SOTA accuracy on most datasets (CT/MRI), high parameter count.
TransUNet	~105.28 M–109 M	~24.6 G–865 G	Hybrid (CNN + Transformer)	High parameter density, superior global context modeling.
Swin-UNet/UNETR	~29.8 M–93 M	~4.45 G–177 G	Pure Transformer (Hierarchical)	Efficient computation (Swin), high performance on 3D patches (UNETR).
MedSAM/SAM-Med3D	>300 M (Image Encoder)	>1000 G (Inference Phase)	Foundation Model (ViT)	Very high cost, zero-shot/prompt capability, challenging on edge devices.

## Data Availability

No data were used in this study.
